# Cytotoxic Effects of Recombinant StxA2-His in the Absence of Its Corresponding B-Subunit

**DOI:** 10.3390/toxins13050307

**Published:** 2021-04-26

**Authors:** Laura Heinisch, Maike Krause, Astrid Roth, Holger Barth, Herbert Schmidt

**Affiliations:** 1 Department of Food Microbiology and Hygiene, Institute of Food Science and Biotechnology, Garbenstraße 28, University of Hohenheim, 70599 Stuttgart, Germany; laura.heinisch@uni-hohenheim.de (L.H.); maike.krause@uni-hohenheim.de (M.K.); astrid.roth.94@web.de (A.R.); 2 Institute of Pharmacology and Toxicology, University of Ulm Medical Center, Albert-Einstein-Allee 11, 89081 Ulm, Germany; holger.barth@uni-ulm.de

**Keywords:** Shiga toxin, Stx2a, subtilase cytotoxin, SubAB, enzyme subunit, cytotoxicity, protein purification strategies, GB_3_ ELISA, Vero B4, HeLa, HCT-116

## Abstract

AB_5_ protein toxins are produced by certain bacterial pathogens and are composed of an enzymatically active A-subunit and a B-subunit pentamer, the latter being responsible for cell receptor recognition, cellular uptake, and transport of the A-subunit into the cytosol of eukaryotic target cells. Two members of the AB_5_ toxin family were described in Shiga toxin-producing *Escherichia coli* (STEC), namely Shiga toxin (Stx) and subtilase cytotoxin (SubAB). The functional paradigm of AB toxins includes the B-subunit being mandatory for the uptake of the toxin into its target cells. Recent studies have shown that this paradigm cannot be maintained for SubAB, since SubA alone was demonstrated to intoxicate human epithelial cells in vitro. In the current study, we raised the hypothesis that this may also be true for the A-subunit of the most clinically relevant Stx-variant, Stx2a. After separate expression and purification, the recombinant Stx2a subunits StxA2a-His and StxB2a-His were applied either alone or in combination in a 1:5 molar ratio to Vero B4, HeLa, and HCT-116 cells. For all cell lines, a cytotoxic effect of StxA2a-His alone was detected. Competition experiments with Stx and SubAB subunits in combination revealed that the intoxication of StxA2a-His was reduced by addition of SubB1-His. This study showed that the enzymatic subunit StxA2a alone was active on different cells and might therefore play a yet unknown role in STEC disease development.

## 1. Introduction

Particular Shiga toxin (Stx)-producing *Escherichia coli* (STEC) strains produce, in addition to Stx, another AB_5_ toxin, the subtilase cytotoxin (SubAB) [1,2]. AB_5_ toxins consist of a catalytic A-subunit and five receptor-binding B-subunits, which form a homopentamer [2]. The production of one or more Stx variants is a major pathogenicity factor of enterohemorrhagic *E. coli* (EHEC). EHEC comprise a STEC subgroup being able to cause gastrointestinal diseases and extraintestinal sequelae such as the life-threatening hemolytic-uremic syndrome (HUS) [3].

Stx belongs to the family of ribosome-inactivating proteins (RIPs) (reviewed in [4]). The A-subunit StxA causes an irreversible removal of an adenine residue from the 28S rRNA in eukaryotic cells [5]. Due to this rRNA *N*-glycosidase activity, protein synthesis is inhibited and apoptosis of the affected cell is initiated [6]. The 32 kD A-subunit is a single polypeptide chain that consists of the two domains A1 and A2, which are inter-connected by a disulfide bond. The 27.5 kD A1-domain exhibits the catalytic activity, whereas the 4.5 kD A2-domain consists of an α-helix, which connects the A-subunit to the B-subunit [1]. The StxB-subunit has a molecular weight of 7.7 kD per monomer [1] and binds mainly to the glycosphingolipid receptor globotriaosylceramide (Gb_3_ or CD77) on the host cell surfaces [4,7,8]. Following receptor recognition, Stx is taken up by endocytosis and transported retrogradely to the Golgi apparatus and subsequently to the endoplasmic reticulum (ER). After processing in the endoplasmic reticulum, the StxA1-domain is translocated into the cytosol to reach the ribosomes (reviewed in [9]).

Stx comprise a family of structurally related toxins, including the two major groups Stx1 and Stx2, each of which is represented by multiple genetic variants. All known Stx variants are encoded in a single operon consisting of *stxA* and *stxB* genes, which are located in lambdoid prophages in the STEC genome [9,10]. Among these, Stx2a is most frequently associated with the development of HUS [11].

The similarly structured subtilase cytotoxin (SubAB) was originally identified in the *E. coli* O113:H21 strain 98NK2, which was isolated from a patient suffering from HUS [12]. The catalytic SubA-subunit is a serine protease which cleaves specifically the ER chaperone BiP/GRP78 [13]. This cleavage leads to an unfolded protein response and eventually apoptosis of target cells [14]. The SubB-subunits bind to the receptor *N*-glycolylneuraminic acid (Neu5Gc) [15], but recent studies indicated other *N*- and *O*-glycans as further target structures for SubB [16]. The SubA-subunit has a molecular weight of approximately 33 kD, whereas SubB has a molecular weight of approximately 14 kD per monomer [17]. Four different genetic variants have been identified for SubAB to date [12,18,19], but more variant genes have been suggested in recent years [20].

Studies on bacterial AB_5_ toxins have demonstrated that the preassembly of the A- and B-subunits to toxin complexes is not essential for cytotoxicity and that the A-subunit alone can exhibit cytotoxic effects [17,21,22]. Funk et al. [22] previously demonstrated such a cytotoxic effect for SubA in Vero B4 cells. Moreover, Pellino et al. [21] have shown that the A and B_5_ preassembly of AB_5_ toxins, by the example of Stx2a, is not required for the in vivo toxicity in mice.

Genes for both, *stx* and *subAB*, can be present in the chromosomes of particular foodborne STEC strains. One of these is *E. coli* O113:H21 strain TS18/08, which was isolated in a previous study [23]. It harbors the *stx_2a_* genes as well as the plasmid-located *subAB_1_* genes [23]. Quantitative real-time PCR (qRT-PCR) experiments showed that both genes were transcribed similarly during laboratory cultivation of this strain, having their transcription optimum after three hours of cultivation [24]. Furthermore, additional qRT-PCR data indicated the role of the global regulators Hfq and H-NS in the regulation of both *stx_2a_* and *subAB_1_* [25].

Bowman et al. [26] demonstrated that hybrid formation of the A-subunit of cholera toxin and the B-subunit of *E. coli* heat-labile toxin is possible. Hybrid toxin formation might have implications for co-infections with different AB_5_ toxin producing strains or infection with strains expressing different AB_5_ toxins in parallel.

In the current study, we hypothesize that the A-subunit of Stx2a alone can intoxicate eukaryotic cells without the corresponding B-subunit. Therefore, separate purification protocols for recombinantly expressed StxA2a-His and StxB2a-His subunits were developed, since common purification strategies mainly focus on the purification of the holotoxin or the separated B-subunit [27,28,29,30]. After identification of the purified subunits by mass spectrometry, the secondary structure and oligomerization of the separate subunits were analyzed using circular dichroism (CD) spectroscopy and size exclusion chromatography, respectively. Separately purified subunits were analyzed alone or in combination in cytotoxicity assays with Vero B4, HeLa, and HCT-116 cells.

Since *subAB* genes have frequently been found in *stx*-positive, *eae*-negative STEC strains, an interaction of both AB_5_ toxin subunits is hypothesized. Therefore, we investigated whether SubA/StxB or SubB/StxA hybrid toxins may be formed. Consequently, recombinants of Stx2a and SubAB1 subunits were used to identify their collaborative cytotoxic potential in cell culture assays. This study aims to give further insight to the cytotoxic activity of Stx2a, unbiased from the paradigm of B-subunit receptor-mediated uptake of the AB_5_ toxin.

## 2. Results

### 2.1. Purification of Stx2a-His Subunits

The genes *stxA_2a_* and *stxB_2a_* were cloned separately in expression vector pET22b(+) as described in the Materials and Methods section. After confirmation of the correct sequence of the recombinant plasmids by Sanger sequencing, expression tests in *E. coli* BL21 (DE3), C41 (DE3), and C43 (DE3) in ZYM-5052, 2YT, and LB broth were performed using different temperatures and incubation times (data not shown). For StxA2a-His, the expression in *E. coli* C43 and auto-induction medium at 37 °C initial growth and 20 °C incubation overnight resulted in the highest protein levels in the soluble fraction of the bacterial cell. After cell lysis, StxA2a-His could be purified using a HisTrap^TM^ column followed by a gel filtration step as described below.

**Figure 1:**
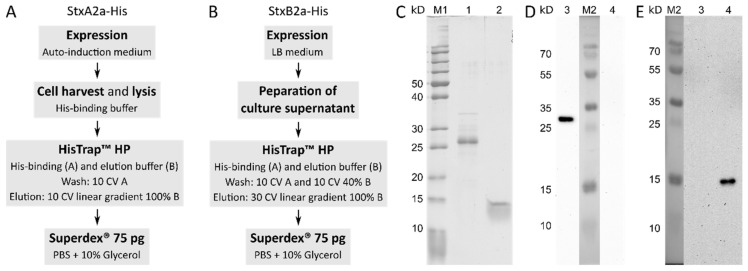
The purification procedures for Stx2a A- and B-subunits are schematically given in (**A**) (StxA2a-His) and (**B**) (StxB2a-His). Gradient elutions are given in column volume (CV). If not otherwise stated, isocratic elutions were applied. Both purifications lead to preparations which show single protein bands in SDS-PAGE, as representatively shown in (**C**). (**C**) PageRuler^TM^ Unstained Protein Ladder (M1; Thermo Fisher Scientific^TM^), 1 µg of StxA2a-His (lane 1), and 1 µg StxB2a-His (lane 2) were applied to 12.5% (*v*/*v*) SDS-PAGE run under reducing conductions. Western blots, given in (**D**,**E**), detected with subunit-specific peptide antibodies Anti-StxA2 and Anti-StxB2, resulted in one band for each purification corresponding to the results of the SDS-PAGE. A total of 500 ng of StxA2a-His (lane 3) and StxB2a-His (lane 4) were subjected to western blot analyses. PageRuler^TM^ Plus Prestained Protein Ladder was used as marker (M2; Thermo Fisher Scientific^TM^).

In contrast, StxB2a-His revealed the highest amount of protein in the culture supernatant when expressed with *E. coli* C41 cultivated in LB broth at initially 37 °C, and 20 °C overnight. After concentration of the culture supernatant, the B-subunit was captured on a HisTrap^TM^ column in the first step and after elution further purified on a Superdex^®^ 75 pg column. A schematic procedure of the expression and purification of the subunits is given in Figure 1A,B.

Representative pools of the protein purification products were applied to a 12.5% (*v*/*v*) SDS-PAGE and are shown in Figure 1C. StxB2a-His preparation was homogenous. StxA2a-His showed some impurities between 30 and 35 kD, which could be identified as *E. coli* proteins. For StxA2a-His and StxB2a-His, one dominant band is visible at approximately 25 kD and close to 15 kD, respectively. The theoretic size of StxA2a-His is 36.8 kD and that of monomeric StxB2a-His is 10.9 kD.

To ensure the purification of the right proteins, preparations of both subunits were submitted to in-gel trypsin digestion (see below). Tryptic peptides were analyzed by Nano-LC-ESI-MS/MS. The obtained spectra of peptides were compared to the protein sequence data base from Uniprot (https://www.Uniprot.org accessed on 1 September 2019) of *E. coli* K12, which was extended by adding Stx2a reference sequences for the A- and B-subunit (StxA2a: NC_002695.1 and StxB2a: NC_002695.1). Both subunits were clearly identified in their respective samples with a total sequence coverage of 63.1% for StxA2a-His and 86.5% for StxB2a-His, respectively. The sequence coverage of both subunits with identified tryptic peptides is depicted in Figure S1. As described above, the StxA2a-His preparation ran at a rather small size of 25 kD compared to expected 36.8 kD on the SDS-PAGE. Although this behavior was unexpected, peptides of all domains of the A-subunit were detected in comparable amounts by mass spectrometry analysis. These findings proved that the subunits were completely expressed and purified, and no degradation of the protein was responsible for the smaller size observed on SDS-PAGE. The observed size of StxB2a-His in the SDS-PAGE is consistent with the literature [28].

### 2.2. Biochemical Characterization of Stx2a Subunits

**Figure 2:**
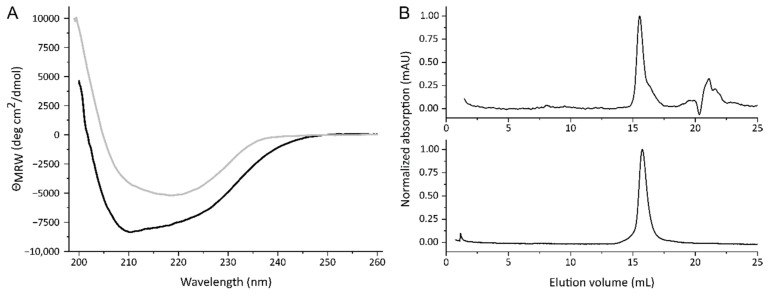
Far-UV spectra (**A**) of StxA2a-His (black curve) and StxB2a-His (grey curve) and size exclusion chromatography (**B**) of StxA2a-His (upper panel) and StxB2a-His (lower panel). All CD and SEC measurements were performed in PBS and with 200 µg/mL samples.

The folding of the purified proteins was assessed by their secondary structure composition. To determine these, samples of the separated Stx2a subunits were evaluated by CD spectroscopy in the far-UV range. Spectra between 260 and 195 nm were recorded and are shown in Figure 2A with the black and grey curves representing StxA2a-His and StxB2a-His, respectively. Based on the crystal structure of Stx2a, the A-subunit should consist of mainly α-helices and have only a minor amount of β-sheets [1]. This is well represented in the far-UV spectrum (see Figure 2A, black curve) with minima at 208 and 220 nm, which are typical for α-helical proteins. The B-subunit of Stx2a, in contrast, contains a large amount of β-sheets. This was also reflected in the CD spectrum (see Figure 2A, grey curve). Here, a minimum at approximately 222 nm and a maximum at 190 nm were expected, due to the rise of the curve towards smaller wavelengths. The quite broad minimum of the StxB2a-His curve also indicates that some part consists of α-helices. Both subunits denatured irreversibly between 50 and 60 °C when heated to 95 °C in temperature transition experiments, obtained by measuring the changes in the CD signal at 218 nm for StxA2a-His and StxB2a-His, respectively (see Figure S3).

Figure 2B shows the typical elution profiles of the separated Stx2a subunits on a Superdex^®^ 200 increase column running in PBS, whereas 100 µL samples of 200 µg/mL were analyzed. Both subunits eluted as one single peak. Based on the calibration of the column (see Figure S3) 36.3 ± 0.3 kD and 32.5 ± 0.6 kD were calculated for StxA2a-His and StxB2a-His, respectively. Compared to the size indicated by SDS-PAGE (see Figure 1C), StxA2a-His revealed the expected molecular weight in the SEC experiments. In contrast, StxB2a-His revealed a much smaller molecular weight than expected. The theoretic molecular weight of a StxB2a-His pentamer should be approximately 53.6 kD, so StxB2a-His appeared smaller in the SEC analyses. To ensure that the B-subunit had a stable oligomerization, samples of 400 and 600 µg/mL were analyzed in addition. No concentration dependent increase in the molecular weight was detectable, indicating that the oligomerization of the B-subunit was stable.

**Figure 3:**
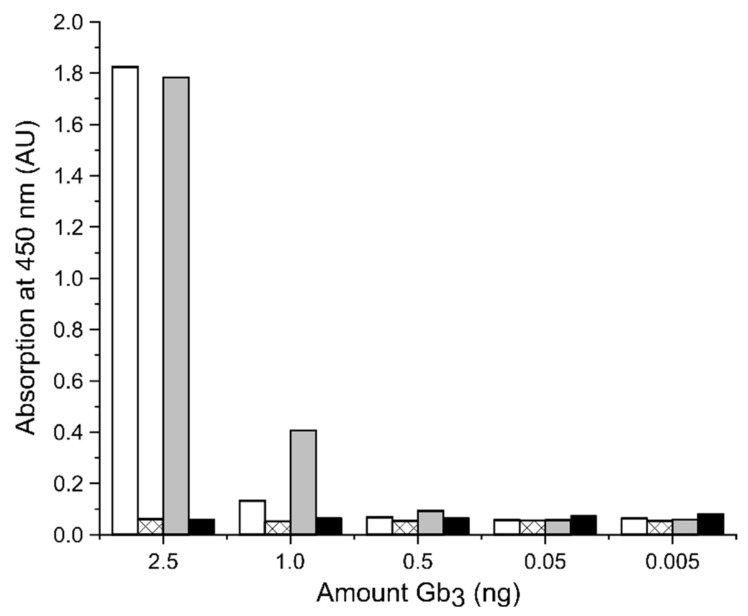
Representative result of the binding of StxB2a-His (white bars), StxA2a-His (crossed bars), and StxAB2a-His (light grey bars) to Gb_3_, detected by ELISA. Wells were coated with different amounts of Gb_3_. A negative control His-tag labeled mBiP was used (black bars). Binding to Gb_3_ was detected at 450 nm through tetramethylbenzidine (TMB) turnover.

To ensure that StxB2a-His was functional in the cytotoxicity assays, a modified Gb_3_ enzyme-linked immunosorbent assay (ELISA) based on Zumbrun et al. [31] was performed. Figure 3 shows the absorption at 450 nm obtained by evaluating the binding of StxB2a-His, StxA2a-His, and a combination of both subunits in a 1:5 molar ratio to decreasing amounts of Gb_3_. This mixture of StxA2a-His and StxB2a-His is designated as StxAB2a-His in the following text. Murine ER chaperone BiP/GRP78 (mBiP) labeled with a His-tag was used as negative control for the detection with anti-His antibody. Less bound StxB2a-His and StxAB2a-His were detected with decreasing amounts of Gb_3_. These results are consistent with previously published data [31]. They prove that StxB2a-His alone is basically functional, and that StxA2a-His alone is not able to bind Gb_3_.

### 2.3. Cytotoxic Effect of StxA2a-His on Different Cell Cultures in the Absence of the B-Subunit

**Figure 4:**
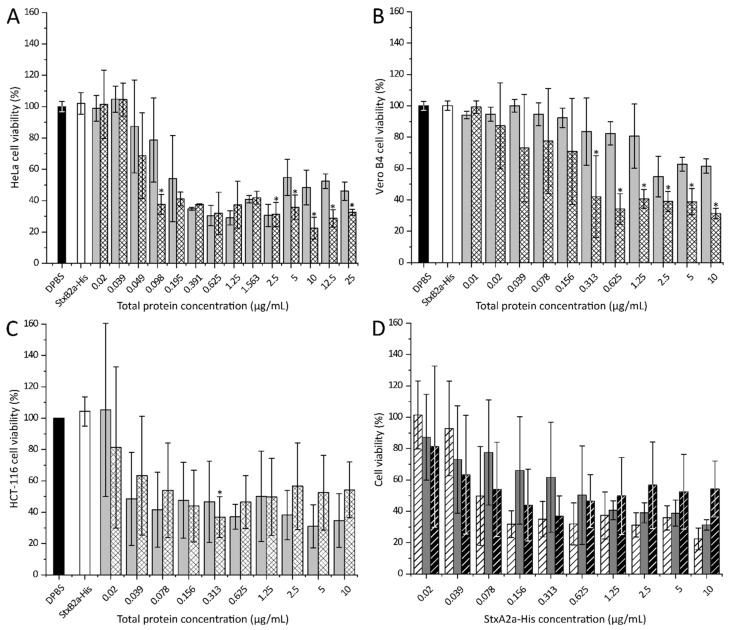
Cytotoxic effects of StxA2a-His on HeLa (**A**), Vero B4 (**B**), and HCT-116 (**C**) cells. Cell viabilities after intoxication with Dulbecco’s phosphate-buffered saline (DPBS, control, black bars), StxB2a-His (white bars), StxA2a-His in a molar ratio of 1:5 with StxB2a-His (StxAB2a-His, light grey bars), and StxA2a-His (crossed bars) are shown. Data of at least three biological replicates are depicted. Cell viability is shown in correlation to total protein concentration and values of DPBS, and StxB2a-His are mean values of all dilutions measured. Comparison of cell viability in presence of StxA2a-His between cell cultures is depicted in (**D**), whereas data of HeLa (dashed white bars), Vero B4 (dark grey bars), and HCT-116 cells (dashed black bars) are shown. Data of at least three biological replicates are depicted. Error bars represent standard deviations, asterisks (*) indicate statistical significance of *p* < 0.05 between StxA2a-His and StxAB2a-His at each toxin concentration for panel (**A**) to (**C**).

To identify in vitro cytotoxic effects of the recombinant A-subunit of Stx2a, each purified subunit, alone or in combination, was applied to different cell cultures. As a control, Dulbecco’s phosphate-buffered saline (DPBS) instead of the toxin solutions was used. After 48 h of intoxication, the amount of attached cells was analyzed by crystal violet staining. Staining intensity was transformed to cell viability using the values of the DPBS-treated samples as 100% viability. Figure 4 shows the cytotoxic effect of StxA2a-His on HeLa, Vero B4, and HCT-116 cells.

As depicted in Figure 4A, the cytotoxic effect of StxA2a-His on HeLa cells (crossed bars) was comparable in strength to the effect observed for the combined subunits (StxAB2a-His, light grey bars) at low total protein concentrations. With increasing total protein concentration, the HeLa cell viability decreased to a minimum of 22 ± 6% when 10 µg/mL StxA2a-His was applied. A significant reduction in cell viability was shown when cells were incubated with StxA2a-His in comparison to cells incubated with StxAB2a-His. As control, StxB2a-His was applied to the cells (white bars), which resulted in no cytotoxic effect after 48 h incubation. Cells treated with 0.02 to 25 µg/mL StxB2a-His showed a comparable amount of attached cells to the control cells (DPBS treated cells, black bars). Independent of the StxB2a-His concentration applied, 100% viable HeLa cells were observed (see Figure S4).

In order to show that the effects of the subunits of Stx2a were not cell-type dependent, the same experiments were performed with Vero B4 and HCT-116 cells (see Figure 4B,C).

A combination of both subunits (StxAB2a-His, light grey bars) showed a cytotoxic effect in a concentration-dependent manner in Vero B4 cells (see Figure 4B). The same effect was observed when StxA2a-His was applied separately (crossed bars), but in this case the cytotoxic effect was more prominent compared to the observations obtained for HeLa cells. At a concentration of 10 µg/mL of StxA2a-His, 31 ± 3% of viable cells was determined. Significant differences in the cytotoxic effect of StxA2a-His compared to StxAB2a-His were observed at concentrations higher than 0.313 µg/mL. Similarly, the progression of the cytotoxic activity curve on HCT-116 (see Figure 4C) shows effects of StxA2a-His and StxAB2a-His. For this cell line, StxA2a-His showed comparable cytotoxic activity to StxAB2a-His and no statistical significance was given. Comparable to the effects observed on HeLa cells, application of StxB2a-His or DPBS to the cells resulted in no effects on the viability of the Vero B4 and HCT-116 cells.

The significantly higher cytotoxic effects of StxA2a-His in comparison to StxAB2a-His on HeLa and Vero B4 cells, normalized to the total protein concentration, should be interpreted with caution (see Figure 4A,B). Since StxA2a-His and StxB2a-His were combined in a 1:5 molar ratio, the concentration of the A-subunit was smaller than in those samples containing StxA2a-His alone. For example, this resulted in 4.03 µg/mL StxA2a-His in the 10 µg/mL total protein concentration of StxAB2a-His. If the cell viability data was normalized to the StxA2a-His concentration, no significant difference in the progress of cytotoxicity was observed between StxA2a-His and StxAB2a-His for all cell lines (see Figure S5). The cytotoxic effect of StxA2a-His alone or in combination with StxB2a-His (StxAB2a-His) showed similar effects on the cell viability of HeLa and HCT-116 cells. Similar cytotoxic effects were observed at lower StxA2a-His concentrations on Vero B4 cells. Higher cytotoxic effects of StxA2a-His without its B-subunit on Vero B4 cells at values of 1.0 to 10 µg/mL StxA2a-His concentration were shown. Similar StxA2a-His concentrations in the StxAB2a-His holotoxin resulted in an increased viability of Vero B4 cells. This effect was not observed for HeLa or HCT-116 cells (Figure 4). The normalization to StxA2a-His concentration verifies the assumption that similar cytotoxic effects were observed for StxA2a-His in the presence or absence of its B-subunit (see Figure S5).

By comparing the cytotoxic effect of StxA2a-His on different cell cultures, a concentration-dependent decrease in cell viability was observed for all cell lines (see Figure 4D). At concentrations of 630 ng/mL StxA2a-His, all cell lines showed a decreased viability by more than 50%. Whereas HeLa (see Figure 4D, dashed white bars) and Vero B4 (see Figure 4D, dark grey bars) showed the minimum cell viability at 10 µg/mL StxA2a-His, the minimum cell viability for HCT-116 cells (see Figure 4D, dashed black bars) was reached already at a toxin concentration of 310 ng/mL StxA2a-His. No significant difference of the cytotoxic activity of StxA2a-His among the cell lines could be detected, which indicates that the cytotoxic effect of StxA2a-His was not dependent on the cell lines tested in this study.

**Figure 5:**
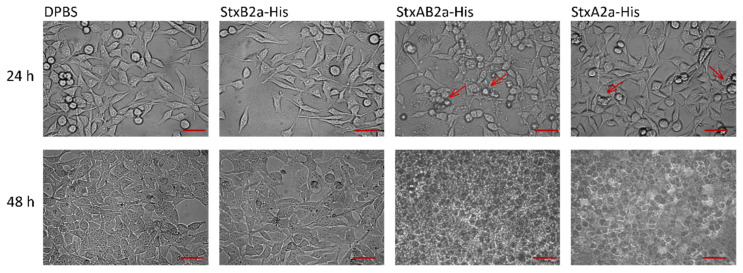
Microscopic analysis of cytotoxic effect of StxA2a-His on HeLa cells. Cells grown in 8-well Nunc permanox chambers and incubated with Dulbecco’s phosphate-buffered saline (DPBS, control), StxB2a-His and StxA2a-His in a molar ratio of 1:5 with StxB2a-His (StxAB2a-His), and StxA2a-His are shown after 24 and 48 h incubation at 37 °C, 5.0% CO_2_. One data set of three biological replicates is shown representatively. Red arrows indicate changes in cell morphology. Scale bar: 50 µm.

To visualize possible differences between the cytotoxic effect of StxA2a-His alone or in combination with StxB2a-His, microscopic analysis was conducted. As shown in Figure 5, no difference in the treatment of HeLa cells with DPBS or StxB2a-His was observed. The cells showed a fibroblast-like morphology with elongated shape. This observation reflects the effects from the cytotoxicity assays.

After 24 h, a changed morphology of cells, such as rounding and irregular shapes, was detected in StxA2a-His- and StxAB2a-His-treated cells (see Figure 5, red arrows). Rounding of the cells resulted in detachment, which can be seen in the results of the cytotoxicity assay by the reduced cell viability. After 48 h, complete lysis of cells was observed in the treated samples. Those effects depicted for HeLa cells were representative for all cell cultures (see Figure S6).

These results show that the cytotoxic effect of StxA2a-His in the absence of its B-subunit can be detected in cell morphology and is consistent with the results described for the crystal violet staining assay of the holotoxin of StxA2a-His and StxB2a-His.

### 2.4. Cytotoxic Effect of StxA2a-His Is Reduced in Presence of SubB1-His

**Figure 6:**
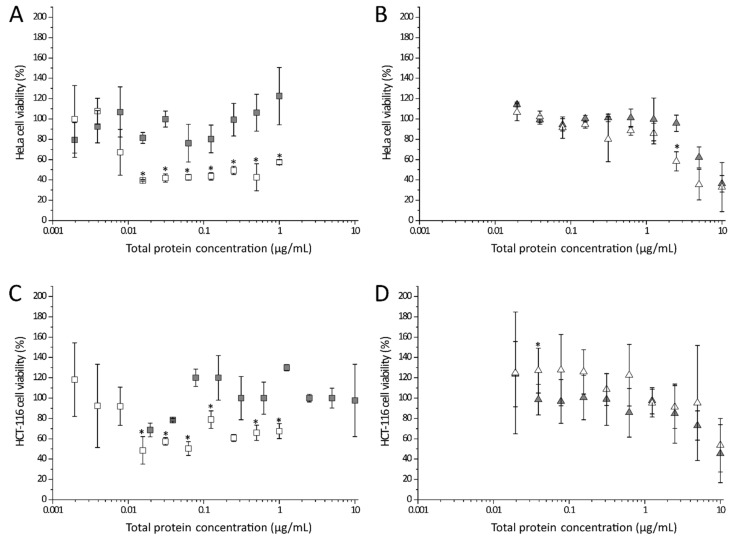
Cytotoxic effect of Stx2a/SubAB1 hybrids on HeLa (upper panels, **A** and **B**) and HCT-116 cells (lower panels, **C** and **D**). Effects of hybrid combination of StxA2a-His and SubB1-His (grey squares, **A** and **C**) or SubA1-His and StxB2a-His (grey triangles, **B** and **D**) in a molar ratio of 1:5 were compared to effects induced by respective A-subunits of Stx2a (white squares) and SubA1 (white triangles). Cells were incubated with StxA2a-His containing samples for 48 h and incubated with SubA1-His containing samples for 72 h. Data of three biological replicates are depicted, error bars show standard deviations, and asterisks (*) indicate statistical significance if *p* < 0.05 between StxA2a-His and StxA2a-His in combination with SubB1-His (panel **A** and **C**) or between SubA1-His and SubA1-His in combination with StxB2a-His (panel **B** and **D**) for each total protein concentration.

We were further interested in the question of whether hybrid toxins can be formed by the two AB_5_ toxins present in STEC. Thus, combinations of StxA2a-His and SubB1-His or SubA1-His and StxB2a-His were investigated for their cytotoxic potential. In Figure 6, the effect of combined Stx2a- and SubAB1-subunits on HeLa (upper panels) and HCT-116 cells (lower panels) is depicted.

The cell viability of HeLa and HCT-116 cells was reduced with increasing StxA2a-His concentration (see Figure 6A,C, white squares) as described above. When samples of StxA2a-His/SubB1-His hybrids were applied to the cells, the cytotoxic effect was significantly reduced compared to the effects measured for StxA2a-His alone. The HeLa cells treated with StxA2a-His/SubB1-His showed only a slight increase in cell viability, showing 122 ± 28% at a total protein concentration of 1 µg/mL. In contrast, intoxication with 1 µg/mL StxA2a-His resulted in 57 ± 2% cell viability (see Figure 6A). The same effect was observed for HCT-116 cells (see Figure 6C). The combination of StxA2a-His with SubB1-His resulted in a reduced cytotoxic effect compared to the cells treated only with StxA2a-His. Incubation with SubA1-His resulted in diminished HeLa cell viability in a concentration-dependent manner. In contrast to the results gained for StxA2a-His in combination with SubB1-His, the addition of StxB2a-His showed no effect on the reduction in the cell viability compared to the treatment with SubA1-His alone (see Figure 6B). In HeLa cells the cytotoxic effect of SubA1-His/StxB2a-His resulted in 36 ± 8% cell viability, whereas SubA1-His alone lead to 32 ± 14% viability at a total protein concentration of 10 µg/mL. No statistically significant difference was given, if SubA1-His was applied separately or in combination with StxB2a-His. The results correlate with the results obtained for HCT-116 cells (see Figure 6D).

## 3. Discussion

In this study, we present a new His-tag-based purification approach for separately cloned and expressed Stx2a subunits. This approach allows a fast purification and has the advantage that a cross-contamination of the A- with the B-subunit and vice versa is excluded. The purified A-subunit showed one dominant band and the B-subunit revealed a single band in SDS-PAGE analysis after purification with Ni-NTA and gel filtration. Those bands were clearly identified by mass spectrometry analyses as StxA2a-His and StxB2a-His, respectively. Western blots with subunit-specific peptide antibodies proved that the A- and B-subunits were exclusively detectable in its corresponding preparation. No signal for the respective adversatively subunit was detected (see Figure 1D,E). The folding and oligomerization were investigated by CD spectroscopy and SEC. StxA2a-His and StxB2a-His revealed the expected composition in the secondary structure (see Figure 2). Fraser et al. [1] crystalized Stx from *S. dysenteriae* and showed that the A-subunit was mainly composed of α-helices. Based on this study, the B-subunit consists of six β-sheets and one α-helix. In the current study, CD spectra indicating a high number of α-helices and β-sheets were detected for Stx2a-His and StxB2a-His, respectively, and were comparable to CD spectra published for Stx2a and StxB2a [30,32].

Determination of the molecular weight revealed the expected 36 kD for StxA2a-His (36.3 kD vs. theoretically 36.8 kD). Nevertheless, the observed smaller band in SDS-PAGE for the A-subunit was rather uncommon for Stx2a [33]. To ensure that the full-length of the A-subunit was applied in the following experiments, mass spectrometry was used to identify peptides of the A1- and A2-domain of StxA2a. Tryptic peptides of StxA2a-His of both domains were found in comparable amounts in all analyses performed. Other studies described a band of 25 kD in their Stx preparation as degraded A-subunit [32], which we can exclude for our preparation, based on the mass spectrometry analysis. However, we assumed that the untypical behavior of StxA2a-His in SDS-PAGE might have been due to an altered ratio of SDS-binding levels to the polypeptide chain [34], which was shown to be dependent on the amino acid sequence [35]. Such observations are in general not unknown for other proteins, but are new for the A-subunit of Stx2a [36].

Contrarily to the results of StxA2a-His, the estimated molecular weight for StxB2a-His based on the SEC analysis was rather small (32.6 kD vs. theoretically 53.6 kD). Conrady et al. [30] performed extensive studies on the oligomerization of the B-subunit for Stx1 and Stx2. They showed by analytical ultracentrifugation that the B-subunit existed in a monomer-pentamer equilibrium. Since different analyzed concentrations of StxB2a-His did not show any changes in the molecular weight of the subunit, we concluded that the oligomerization was stable. Already, other B-subunits of AB_5_ toxins showed smaller molecular weight, as expected after SEC analysis [17]. This could be due to a more compact folding of the B-subunit. StxB2a-His and StxAB2a-His were able to bind Gb_3_, which was shown by a Gb_3_ ELISA (see Figure 3), and showed a comparable behavior to that published for other Stx holotoxin preparations [31].

Purified Stx2a-subunits were used for cytotoxicity assays. In those assays, StxA2a-His, separately or in combination with StxB2a-His (StxAB2a-His), showed cytotoxic activity. The cytotoxic activity observed for the StxA2a-subunit was comparable to the cytotoxicity measured when the combined recombinant StxAB2a-holotoxin was applied to HeLa and HCT-116 cells. The overall achieved cytotoxicity for StxA2a-His and StxAB2a-His was rather low compared to values stated in the literature [37,38]. Nevertheless, control experiments with purified Stx2a holotoxin revealed comparable HeLa cell viabilities to the reconstituted StxAB2a-His holotoxin under our experimental settings. When applied to Vero B4 cells, StxA2a-His showed even higher cytotoxic effects in the absence of its B-subunit compared to the reconstituted holotoxin StxAB2a-His. StxB2a-His itself showed no cytotoxic activity at all, and the cell viability of all analyzed cell cultures was comparable to the control samples (see Figure 4). The results in this study demonstrate the effect of StxA2a-His on HeLa, Vero B4, and HCT-116 cells independent of its B-subunit. Interestingly, the cytotoxic activity of StxA2a-His did not vary between the investigated cell lines and, moreover, no difference in cytotoxic activity between StxA2a-His and StxAB2a-His was observed in HeLa and HCT-116 cells. These results were verified by microscopic analysis, whereas morphological changes in the cell structure were observed after 24 h of incubation. Both treatments with StxA2a-His alone or in combination with its B-subunit showed differences in morphology compared to cells incubated with DPBS or StxB2a-His. The morphological changes observed in the cells with StxA2a-His or StxAB2a-His could not be differentiated. Microscopic analysis verified the apoptotic effect of StxA2a-His in HeLa, Vero B4, and HCT-116 cells.

To our knowledge, the cytotoxicity of the StxA2a-subunit independent of its B-subunit is uniquely described. Thus, we recommend designating this effect as the single-A effect. Funk et al. [22] described a similar effect for SubA on HeLa cells, whereas the cytotoxicity of SubA1 was seen at higher concentrations than the combined holotoxin. Comparable to the results seen in our study, the B-subunit of the AB_5_ toxin did not exhibit any cytotoxicity if applied separately to HeLa cells [22].

The results described in this study show that the A-subunit of Stx can exhibit cytotoxic activity independent of the B-subunit. This leaves the question of the mechanism of binding, uptake, and cytotoxicity of the A-subunit without the presence of the B-subunit. Studies have indicated the importance of StxA on the endocytosis process. Torgersen et al. [39] showed that endocytosis of Stx in HeLa and Vero cells, amongst others, was triggered by the surface-bound A-subunit in the holotoxin. In contrast, the binding of the B-subunit to the receptor did not induce endocytosis [39]. Besides the impact of the A-subunit in the holotoxin on endocytosis, a recent study suggested that Stx is not released as a holotoxin and AB_5_ pre-assembly is formed at the cell surface [21]. Sessler et al. demonstrated that the A-subunit of AB_5_ toxins, such as SubA2-2, can be taken up and transported to the endoplasmic reticulum without its B-subunit [40].

Besides the receptor-mediated endocytosis of Stx, several studies demonstrated an alternative method of uptake. It was shown that toxins could be secreted from *E. coli* O157:H7 cells in outer membrane vesicles (OMVs) [41,42,43]. Moreover, Bielaszewska et al. [44] showed that OMVs are formed and released at the bacterial cell membrane containing multiple virulence factors and uptake at the host cell functions via dynamin-dependent endocytosis. Inside the cell, Stx2a is separated from the OMVs in the early endosomes and is retrogradely transported to the Golgi complex and further to the endoplasmic reticulum [44]. Furthermore, Kim et al. [45] demonstrated that the StxB-subunit can be transported with OMVs in an *E. coli* O157:H7 ∆*stxA* deletion mutant, indicating that even separated subunits can be transported by this pathway. Thus, we suggest that the subunits of Stx2a, and in particular StxA2a, can be transported separately from the other subunit and even probably independent of the classical Gb_3_-binding by pathways such as transport via outer membrane vesicles [45].

Experiments using hybrid combinations of recombinant subunits of Stx and SubAB showed that the cytotoxic effect of StxA2a-His on HeLa and HCT-116 cells is reduced in the presence of SubB1-His (see Figure 6). The B-subunit of SubAB is known to bind to the receptor *N*-glycolyneuraminic acid [15], but recent studies have demonstrated binding to all *N*-glycans presented on the cell surface [16]. Thus, we assume that SubB1-His may bind to the glycosylated structures blocking the target structure for StxA2a-His uptake. In contrast, SubA1-His cytotoxicity was not reduced in combination with StxB2a-His. In this study, we could reproduce the cytotoxic effect of SubA1-His described by Funk et al. [22], whereas SubA1-His exhibited a single-A cytotoxic activity on HeLa and HCT-116 cells. This effect was not reduced by the presence of StxB2a-His. It is known that StxB2a binds specifically to the glycosphingolipid globotriaosylceramide (Gb_3_) [7]. Thus, if there was a specific target structure for SubA1 on the cell surface, this would not be blocked due the binding of StxB2a-His, assuming that Gb_3_ is not the target structure for SubA1-His.

Several studies have indicated the importance of Gb_3_ presence on Stx cytotoxicity in the absence of the receptor-mediated B-subunit. The inhibition of the ceramide glucosyltransferase leading to less Gb_3_ presented on the surface of HeLa and Vero cells lead to a protection from cytotoxic effects induced by Stx1 [46]. Furthermore, it could be shown that even in cell culture free systems the binding of Stx2a to the receptor Gb_3_ is needed for cytotoxicity induction. Johansson et al. [47] showed in extracellular vesicles that the presence of Gb_3_ on the recipient cell is important for Stx-induced change in metabolism or protein translation. Uptake of microvesicles containing Stx2a did not induce changes in metabolism when Gb_3_ was reduced on the cell surface of HeLa cells [47]. To the contrary, a study of Schüller et al. [48] demonstrated on in vitro organ culture systems that the human intestinal epithelium showed Stx2-induced damage even in the absence of Gb_3_ receptors, which indicates an alternative uptake/transport mechanism for Stx2a cytotoxicity. We conclude that the uptake of StxA2a-His is mediated by a target structure presented on the cell surface which is probably not Gb_3,_ since binding of StxA2a-His in the Gb_3_-ELISA was not observed (see Figure 3).

Although we described the cytotoxic activity, the mechanism by which StxA2a-His is taken up and transported inside the cell remains open and will be the subject of further studies.

## 4. Conclusions

In the present study, we developed purification strategies for both Stx2a subunits as recombinant proteins, based on affinity chromatography. By mass spectrometry, CD spectrometry, and SEC the expected biochemical characteristics of the respective subunits were analyzed. Moreover, purified subunits alone or a combination with a 1:5 molar ratio of both subunits were applied in cytotoxicity assays using HeLa, Vero B4, and HCT-116 cells. StxA2a-His exhibited a cytotoxic effect in the absence of its B-subunit, an effect that we have designated the single-A effect. This effect was independent of the cell lines investigated. Whereas this study is the first to describe a single-A effect of Stx2a, no conclusion according to the uptake mechanism or intracellular processes induced in the cell metabolism can be drawn. Further research is needed to explore the mechanisms of binding, uptake, and intracellular transport to elucidate this single-A mechanism of StxA2a further.

## 5. Material and Methods

### 5.1. Cloning and Expression Tests of Recombinant Stx2a Subunits

To clone the *stx2a* subunit genes, genomic DNA (gDNA) of *E. coli* DH5α/933W (see Table 1) was prepared. Thus, a DNeasy Blood and Tissue kit (Qiagen, Hilden, Germany) was used and gDNA was prepared as described in the protocol provided by the manufacturer using 4 mL of an overnight culture of *E. coli* DH5α/933W (DNeasy Blood and Tissue handbook, July 2006; Qiagen, Hilden, Germany). For amplification of *stxA2a* and *stxB2a*, the purified gDNA and primers StxA2a-pET22_for, StxA2a-pET22_rev, StxB2a-pET22_for, and StxB2a-pET22_rev containing restriction sites for *Nde*I and *Xho*I were used (see Table 2).

Plasmids were prepared using a QIAprep spin miniprep kit (Qiagen, Hilden, Germany) by following the manufacturer’s recommendations using 2 mL of the respective overnight cultures (QIAprep miniprep handbook from July 2006; Qiagen, Hilden, Germany). Purity and concentration of nucleic acids were determined spectrophotometrically using a NanoDrop 2000 device (Thermo Fisher Scientific, Waltham, MA, USA).

PCR products of *stxA2a* and *stxB2a* genes and the cloning vector pET22b(+) were digested with *Xho*I (Thermo Fisher Scientific, Waltham, MA, USA) and *Nde*I (Thermo Fisher Scientific, Waltham, MA, USA) in 2× Tango buffer (Thermo Fisher Scientific, Waltham, MA, USA). For the double digest, 1.0 µg of DNA in a total sample volume of 20 µL was incubated at 37 °C for one hour and the restriction enzymes were then inactivated at 80 °C for 20 min. Restricted samples were purified by a QIAquick PCR purification kit following the manufacturer’s recommendations (QIAquick spin handbook, April 2015; Qiagen, Hilden, Germany). The *stxA2a* and *stxB2a* PCR products and the vector were ligated using a molar ratio of vector to insert of 1:5, using 10 µg vector, and T4 DNA ligase (Thermo Fisher Scientific, Waltham, MA, USA). The ligation was conducted at 22 °C for one hour. Subsequently, ligation batches were transformed to chemically competent *E. coli* DH5α as described previously [25].

**Figure 7:**
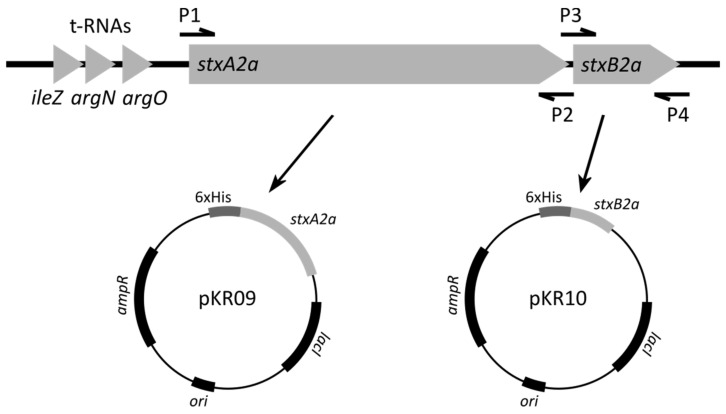
Schematic depiction of the principle of cloning of the stx2a subunits. In the upper part the 933W phage DNA with the binding sites of the primers is shown. P1 refers to the primer StxA2a-pET22_for, P2 to primer StxA2a-pET22_rev, P3 represents primer StxB2a-pET22_for, and P4 primer StxB2a-pET22_rev. A schema of the expression plasmids pKR09 and pKR10 are depicted in the lower row. Besides the position of C-terminal His-Tag (6xHis), the position of the ampicillin resistance gene (ampR) as well as of the lac repressor (lacI) is indicated.

To verify constructed plasmids, they were sequenced by Sanger sequencing using primers pET22-seq-for and pET22-seq-rev (see Table 2) as described earlier [51]. After confirmation of the correct insertion of the *stxA2a* and *stxB2a* genes in the pET22b(+) expression vector, plasmids were transformed to chemically competent *E. coli* C41 (DE3) or competent *E. coli* C43 (DE3) cells, respectively, as described earlier [25]. The expression plasmids for *stxA2a* and *stxB2a* were designated pKR09 and pKR10, respectively. An overview of the cloning procedure is provided in Figure 7.

Expression tests for both constructs in *E coli* BL21 (DE3), C41 (DE3), and C43 (DE3) were conducted in LB broth, 2YT broth, and auto-induction medium ZYM-5052 without trace elements as described in [52] (400 mL of ZYM-5052 consisted of 200 mL of two-fold concentrated ZY (2% [*w*/*v*] tryptone, 1% [*w*/*v*] yeast extract, pH 7.0), 8 mL of 50× M (1.25 mol/L Na_2_HPO4, 1.25 mol/L KH_2_PO_4_, 2.5 mol/L NH_4_Cl, 0.25 mol/L Na_2_SO_4_), 8 mL of 50× 5052 (2.5% [*w*/*v*] glycerol, 0.25% [*w*/*v*] D-(+)-glucose, 1.0% [*w*/*v*] lactose), and 0.8 mL of 1 mol/L MgSO_4_), respectively. Initial growth temperatures of 37, 25, and 20 °C until induction and 4 h or overnight expression at 25 and 20 °C were applied to increase the subunit expression in the soluble fraction of the bacterial cell pellet or the culture supernatant.

### 5.2. Recombinant Expression and Purification of Toxin Subunits

The recombinant StxA2a-His subunit was expressed using *E. coli* C43 (DE3)/pKR09 in auto-induction medium ZYM-5052 without trace elements. The expression cultures were supplemented with 150 µg/mL ampicillin and inoculated 1:50 with an LB overnight culture containing 150 µg/mL ampicillin (37 °C, 180 rpm) of the expression strain. After an initial incubation at 37 °C and 180 rpm until an optical density at 600 nm (OD_600_) of 1.3–1.5, the temperature was decreased to 20 °C and the culture further incubated for 20 h. After expression, the cells were harvested at 5000× *g*, 4 °C for 15 min and washed with 30 mL phosphate-buffered saline (PBS). Cell lysis was conducted by resuspending the cell pellet in His-binding buffer (50 mmol/L Tris, 300 mmol/L NaCl, pH 8.0) using 5 mL buffer per 1 g cells, the addition of 1 mg/mL lysozyme, and incubation for 1 h on ice. Cell disruption was carried out by ultrasonication using 12 cycles of ultrasonic steps for 10 s each with 20 s cooling on ice in between (Sonifier^®^ SFX150 equipped with a microtip, Branson Ultrasonic Corporation, Danbury, CT, USA). The insoluble fraction was separated by centrifugation 25,000× *g*, 4 °C for 45 min (Avanti J25 centrifuge, Beckman Coulter, Brea, CA, USA). For the purification of StxA2a-His, the cleared lysate was loaded on a 5 mL HisTrap^TM^ HP column (GE Healthcare, Chicago, IL, USA) equilibrated in His-binding buffer. To remove all unbound components, the column was washed with 6 column volumes (CV) His-binding buffer. Elution was performed with a linear gradient from 0 to 100% of elution buffer (50 mmol/L Tris, 300 mmol/L NaCl, 250 mmol/L imidazole, pH 8.0) over 12 CV. Sodium dodecyl sulphate polyacrylamide gel electrophoresis (SDS-PAGE) as described previously [53] and stained by Quickstain Coomassie^®^ stain solution (SERVA Electrophoresis GmbH, Heidelberg, Germany), as well as western blots, were used to identify StxA2a-His containing fractions. Therefore, SDS-PAGE gels were blotted on a polyvinylidene fluoride (PVDF) immunoblot membrane (Bio-Rad Laboratories Inc., Hercules, CA, USA). Stx2a subunits were detected by synthesized specific primary peptide antibodies Anti-StxA2 and anti-StxB2 (gifted by Alexander Mellmann and Petya Berger, University of Münster, Germany), and a horseradish conjugated secondary antibody (goat anti-rabbit IgG (H+L) secondary antibody, goat/IgG; Thermo Fisher Scientific, Waltham, MA, USA). Detection was performed by chemiluminescence (ECL system) and a BioRad Chemidoc XRS+ device (Bio-Rad Laboratories Inc., Hercules, CA, USA). Positive fractions were subsequently pooled and concentrated using an Amicon Ultra Centrifugal Filter (molecular weight cut off of 3000 Da, Merck Millipore, Germany) following the manufacturer’s recommendations. Remaining impurities were removed by a size exclusion chromatography step performed on a Superdex^®^ 75 pg 16/600 (GE Healthcare, Chicago, IL, Chicago, IL, USA) in PBS supplemented with 10% (*v*/*v*) glycerol. After sample application the isocratic elution was collected in 2 mL fractions. Again, StxA2a-His-containing fractions were identified with SDS-PAGE followed by western blot and pooled. After concentration and detection of the protein concentration, StxA2a-His was aliquoted in 100 µL portions and stored at −70 °C until further usage.

The recombinant expression of StxB2a-His was conducted with *E. coli* C41 (DE3)/pKR10 in LB broth supplemented with 150 µg/mL ampicillin. A 400 mL expression culture was inoculated with an overnight culture as described above and incubated at 37 °C and 180 rpm. At an OD_600_ of 0.4 the expression was induced by the addition of 250 µmol/L isopropyl β-D-1-thiogalactopyranoside (IPTG) and the temperature subsequently reduced to 20 °C. After 20 h further incubation the cells were harvested as described above and the supernatant used for further processing. The culture supernatant was filtered with a 0.2 µm Nalgene filter (Nalgene, USA) and concentrated to less than 50 mL by cross flow using a Vivaflow200 ultrafiltration module (Sartorius Stedim Lab LTD, Winterbach, Germany). StxB2a-His was purified in a two-column approach consisting of an affinity chromatography and a size-exclusion chromatography step operated on an ÄKTA pure system. First, the concentrated supernatant was loaded on a HisTrap^TM^ HP column. After washing of the column with 6 CV His-binding buffer, impurities were removed by a second wash step of 10 CV with 40% elution buffer. StxB2a-His was eluted with a linear gradient from 40 to 100% elution buffer in 10 CV and 3 mL fractionation. Fractions containing StxB2a-His were identified via SDS-PAGE and western blot as described above and subjected to a Superdex^®^ 75 pg running in PBS supplemented with 10% (*v*/*v*) glycerol as described before. Pure StxB2a-His fractions were pooled, concentrated, and stored in 100 µL aliquots at −70 °C.

Purified Stx subunits were identified after tryptic digestion of protein bands excised from SDS-PAGE using Nano-LC-ESI-MS/MS performed on an Ultimate 3000 RSLCnano system (Dionex, Thermo Fisher Scientific, Waltham, MA, USA) coupled to a Q-Exactive HF-X mass spectrometer (Thermo Fisher Scientific, Waltham, MA, USA) using an EASY-Nano Flex ion source (Thermo Fisher Scientific, Waltham, MA, USA) from the mass spectrometry core facility of the University of Hohenheim.

The SubAB subunits were recombinantly expressed and purified as previously described [17].

### 5.3. Determination of Protein Concentration

Protein concentrations were determined spectrophotometrically using a NanoDrop^®^ 2000 device (Thermo Fisher Scientific, Waltham, MA, USA). Therefore, absorption was measured at 280 nm and the concentrations were calculated by using the respective theoretical extinction coefficient and molecular mass ascertained by ProtParam (see Table 3). Due to the low extinction coefficient of StxA2a-His, the concentration was determined by a Bradford assay as previously described [54]. The Bio-Rad protein assay dye (Bio-Rad Laboratories Inc., Hercules, CA, USA) was used and a standard curve using bovine serum albumin (BSA, Carl Roth GmbH + Co.KG, Karlsruhe, Germany) was performed. Detection was conducted at 595 nm using a Tecan Infinite M200 device (Tecan, Männedorf, Switzerland).

### 5.4. CD Spectroscopy

Far-UV spectra were measured from 260 to 195 nm at 25 °C with 100 nm/min of continuous scanning, 0.1 nm of data pitch, 4 s of response time, 1 nm of band width, and 10 accumulations at a Jasco J715 CD Spectrometer with a PTC-348 WI Peltier Unit using default settings (Jasco, Pfungstadt, Germany). Raw data was recorded in millidegrees and the signal was converted to the mean residue ellipticity (deg∙cm^2^/dmol). Prior to the measurements, all samples were dialyzed in standard PBS buffer overnight and the concentration adjusted to 200 µg/mL for each sample. Denaturation curves were recorded at 220 nm for StxA2a-His and 218 nm for StxB2a-His with 1 °C/min of heating rate, 0.1 °C of data pitch, 1 s of response time, and 1 nm of band width. Denaturation temperatures were estimated with simple logistic fits.

### 5.5. Size Exclusion Chromatography

For an estimation of the molecular size and oligomerization of the subunits, a Superdex^®^ 200 increase 10/30 GL column (GE Healthcare, Chicago, IL, USA) was calibrated with two sets of calibration proteins, consisting of thyroglobulin, aldolase, ovalbumin, and ribonuclease A and ferritin, conalbumin, carbonic anhydrase, and aprotinin following the manufacture’s recommendation (GE Healthcare HMW/LMW calibration kit, GE Healthcare, Chicago, IL, USA). The void volume was detected with Blue dextran. The molecular weight was calculated based on a logarithmic fit by the determination of the *K_av_* value. All measurements were performed in PBS and at least thrice of protein samples with 200 µg/mL concentration.

### 5.6. Gb_3_ ELISA

Gb_3_ binding was analyzed by a modified ELISA as described by Zumbrun et al. [31]. Briefly, 2HB 96-well plates (Thermo Fisher Scientific, Waltham, MA, USA) were coated with Gb_3_ (Matreya LLC, State College, PA, USA) suspended in 96% EtOH p.A. overnight and washed with 100 µL PBST (PBS, 0.05% (*v*/*v*) Tween 20) for 1 h at 4 °C. After blocking with 200 µL PBST + 3% (*w*/*v*) BSA for 2 h at 4 °C and washing twice with 150 µL PBST + 0.2% (*w*/*v*) BSA (PBST/0.2% BSA), 20 ng protein in 100 µL PBST/0.2% BSA was applied to each well and incubated for 1 h at room temperature. For the detection, each well was incubated with 100 µL 6×-His tag monoclonal antibody (His.H8, Thermo Fisher Scientific, Waltham, MA, USA) diluted 1:3000 in PBS after washing the wells thrice with 200 µL PBST/0.2% BSA. After another three times washing with 200 µL PBST/0.2% BSA, goat anti-mouse IgG (H+L) conjugated to horseradish peroxidase (Thermo Fisher Scientific, Waltham, MA, USA) was applied in a 1:2000 dilution in 100 µL PBS as secondary antibody and incubated for 1 h at room temperature. The detection was performed at 450 nm with tetramethylbenzidine (TMB) peroxidase substrate solution (Bio-Rad Laboratories Inc., Hercules, CA, USA) according to the manufacturer’s recommendation. The ELISA was performed with StxA2a-His, StxB2a-His, and a 1:5 molar combination of the two subunits (StxAB2a-His). His-tag labeled murine ER-specific chaperone BiP/GRP78 (mBiP) was used as a negative control. The experiment was conducted in three independent biological replicates.

### 5.7. Cytotoxicity Assays

Cytotoxic activity assays were conducted as described previously [22]. Briefly, human cervix cancer-derived epithelial cell line HeLa cells, human colon carcinoma-derived HCT-116 cells (DMSZ No. ACC581), or Vero B4 cells (DMSZ No. ACC 33) were used. HeLa cells were grown in MEM medium (Gibco^®^, Thermo Fisher Scientific, Waltham, MA, USA) supplemented with 10% (*v*/*v*) heat-inactivated fetal calf serum (FCS), 1% (*v*/*v*) non-essential amino acid solution (Gibco^®^, Thermo Fisher Scientific, Waltham, MA, USA), 1% (*v*/*v*) sodium pyruvate (Thermo Fisher Scientific, Waltham, MA, USA), and 2 mmol/L glutamine (Thermo Fisher Scientific, Waltham, MA, USA). HCT-116 cells were grown in DMEM medium containing sodium pyruvate (Gibco^®^, Thermo Fisher Scientific, Waltham, MA, USA) supplemented with 10% (*v*/*v*) heat-inactivated fetal calf serum (FCS), 1% (*v*/*v*) non-essential amino acid solution (Gibco^®^, Thermo Fisher Scientific, Waltham, MA, USA), and 2 mmol/L glutamine (Thermo Fisher Scientific, Waltham, MA, USA). Vero B4 cells were cultivated in RPMI 1640 medium (Gibco^®^, Thermo Fisher Scientific, Waltham, MA, USA) supplemented with 10% (*v*/*v*) heat-inactivated fetal calf serum (FCS). All cultures were passaged by trypsinization and incubated at 37 °C, 5.0% CO_2_. Cells were used in passages 5 to 30. For cytotoxicity assays, the cells were seeded in a 96-well chamber at a concentration of 1.0 × 10^4^ cells/well and incubated at 37 °C, 5.0% CO_2_ for 24 h. After washing of the cells, subunit solutions were applied in serial dilutions in the respective medium. For intoxication with Stx2a-subunits, incubation times of 48 and 72 h were used when SubAB1-subunits were applied [22]. Analysis of the assay was performed using crystal violet staining and detection at 570 nm using a Tecan Infinite M200 microtiter plate reader (Tecan, Männedorf, Switzerland). All experiments were conducted in three technical replicates and three independent biological replicates. Cell viability was calculated using the optical density of the control-treated samples, incubated with Dulbecco’s phosphate-buffered saline (DPBS), as 100% cell viability.

### 5.8. Microscopic Analysis

To investigate cytotoxic effects of the Stx2a-subunits on HeLa, Vero B4, and HCT-116 cells, Nunc^®^ permanox 8-well chambers (Thermo Fisher Scientific, Waltham, MA, USA) were used. Cell cultures were grown and passaged as described above. For analysis, 1.0 × 10^4^ cells/well were seeded in the 8-well chamber at a total volume of 400 µL/well. Cells were incubated at 37 °C, 5.0% CO_2_ for 24 h. For intoxication, each well was washed with 400 µL DPBS and cell type-dependent fresh medium was added to the cells. Cells were incubated with 0.5 µg/mL total protein concentration and incubated further at 37 °C, 5.0% CO_2_. After 24 and 48 h cells were analyzed by microscopy using an inverted microscope Axio Vert.A1 (Zeiss, Oberkochen, Germany) equipped with a color camera (Axiocam 105, Zeiss, Oberkochen, Germany). Pictures were detected using a 40×/1.25 objective (Zeiss, Oberkochen, Germany) and processed by the program ZEN light (Zeiss, Oberkochen, Germany). Experiments were conducted as two technical replicates in at least three biological replicates.

### 5.9. Statistical Analysis

If not stated differently, all data were analyzed using OriginPro2020 from OriginLab, USA. For statistical analysis of cytotoxic effects, data of cell viability of all biological replicates were used. Initially, data were analyzed on a normal distribution. If the data set was drawn from a normally distributed population, two data sets were compared using the two-tailed Student’s *t*-test. Variances of data sets were analyzed with Students *t*-test on variances. Welch’s *t*-test was applied to not-normally distributed data sets and if equal variances were not given. Effects compared between all cell cultures were analyzed using one-way analysis of variance (ANOVA) followed by post-hoc Tukey test. Statistical significance was given at values of *p* smaller than 0.05.

## Figures and Tables

**Table 1:** Strains and plasmids used in this study.

Strain or Plasmid	Relevant Geno- or Phenotype	Reference
*E. coli* DH5α	*tonA lacZ∆*M15 *endA1 recA1 thi-1 supE44 phoA gyrA96 hsdR17 Δ(lacZYA-argF)U169 relA1*	Invitrogen
*E. coli* DH5α/933W	DH5α strain carrying the Stx2a-encoding prophage 933W	This study
*E. coli* BL21 (DE3)	Expression strain, *dcm ompT hsdS*(r_B_^−^m_B_^−^) *gal*	[49]
*E. coli* C41 (DE3)	Expression strain derived from *E. coli* BL21, T7 promoter driven expression, *lac*I operon	[50]
*E. coli* C43 (DE3)	Expression strain derived from *E. coli* BL21, T7 promoter driven expression, *lac*I operon	[50]
*E. coli* C43 (DE3)/pKR09	Expression strain expressing StxA2a-His subunit	This study
*E. coli* C41 (DE3)/pKR10	Expression strain expressing StxB2a-His subunit	This study
pET22b(+)	Amp^R^, 6x His-tag	Novagen Inc.
pKR09	pET22b(+)-*stxA2a*, Amp^R^	This study
pKR10	pET22b(+)-*stxB2a*, Amp^R^	This study

**Table 2:** Oligonucleotide primers used in this study.

Primer Designation	Sequence (5′ to 3′)	Source
StxA2a-pET22_for	CGTGCATATGAAGTGTATATTATTTAAATGGGT	This study
StxA2a-pET22_rev	CCCCTCGAGTTTACCCGTTGTATATAA	This study
StxB2a-pET22_for	CGTGCATATGAAGAAGATGTTTATGGCGG	This study
StxB2a-pET22_rev	CCCCTCGAGGTCATTATTAAACTGCAC	This study
pET-22b-seq-for	GGGTTATGCTAGTTATTGC	This study
pET-22b-seq-rev	GCGAAATTAATACGACTCAC	This study

* underlined letters indicate restriction sites.

**Table 3:** Molecular weight and extinction coefficients of toxin subunits used in this study. Parameters were calculated based on the amino acid sequence with the online tool ProtParam.

Toxin Subunit	Molecular Weight /kDa	Extinction Coefficient /M^−1^cm^−1^
StxA2a-His	36.8	33,140
StxB2a-His	10.9	13,980
SubA1-His	36.1	41,035
SubB1-His	14.0	26,930

## Data Availability

Data is contained within the article or Supplementary Materials.

## Supplementary Materials

The following are available online at https://www.mdpi.com/article/10.3390/toxins13050307/s1, Figure S1: Sequence coverage of StxA2a-His (A) and StxB2a-His (B) based on the mass spectrometry analysis, Figure S2: Thermal denaturation curves of StxA2a-His (A) and StcB2a-His (B), Figure S3: Logarithmic calibration curve for the Superdex^®^ 200 increase column for the evaluation of the size exclusion experiments, Figure S4: Cytotoxic effect of StxB2a-His on HeLa (A), Vero B4 (B), and HCT-116 (C) cells, Figure S5: Cytotoxic effect of StxA2a-His on HeLa (A), Vero B4 (B), and HCT-116 (C) cells, Figure S6: Microscopic analysis of cytotoxic effect of StxA2a-His on HeLa, Vero B4, and HCT-116 cells.
