# Supplementary material for: Cytotoxic Effects of Recombinant StxA2-His in the Absence of Its Corresponding B-Subunit
Source: Toxins (Basel). 2021 Apr 26;13(5):307. doi: 10.3390/toxins13050307 (PMC8145687; doi:10.3390/toxins13050307)
Supplement: Supplementary file 1 [file toxins-13-00307-s001.zip › toxins-1161408-supplementary.pdf]

Supplementary Materials: Cytotoxic Effects of Recombinant StxA2-His in Absence of its Corresponding B-Subunit

Laura Heinisch, Maike Krause, Astrid Roth, Holger Barth and Herbert Schmidt

A

```
        10         20         30         40         50         60
MKCILFKWVL CLLLGFSSVS YSREFTIDFS TQQSYVSSLN SIRTEISTPL EHISQGTTSV
        70         80         90        100        110        120
SVINHTPPGS YFAVDIRGLD VYQARFDHLR LIIEQNNLYV AGFVNTATNT FYRFSDFTHI
       130        140        150        160        170        180
SVPGVTTVSM TTDSSYTTLQ RVAALERSGM QISRHSLVSS YLALMEFSGN TMTRDASRAV
       190        200        210        220        230        240
LRFVTVTAEA LRFRQIQREF RQALSETAPV YTMTPGDVDL TLNWGRISNV LPEYRGEDGV
       250        260        270        280        290        300
RVGRISFNNI SAILGTVAVI LNCHHQGARS VRAVNEESQP ECQITGDRPV IKINNTLWES
       310        310
NTAAAFLNRK SQFLYTTGKL EHHHHHH
```

B

```
        10         20         30         40         50         60
MKKMFMAVLF ALASVNAMAA DCAKGKIEFS KYNEDDTFTV KVDGKEYWTS RWNLQPLLQS
        70         80         90
AQLTGMTVTI KSSTCESGSG FAEVQFNNDLE HHHHHH
```

Figure S1. Sequence coverage of StxA2a-His (**A**) and StxB2a-His (**B**) based on the mass spectrometry analysis. StxA2a-His has a maximal coverage of 61.3% and StxB2a-His of 86.5%. Found tryptic peptides are highlighted in grey. Additional amino acids which are not included in the reference sequences (StxA2a: NC_002695.1 and StxB2a: NC_002695.1) and belong to the His-tag are written in bold.

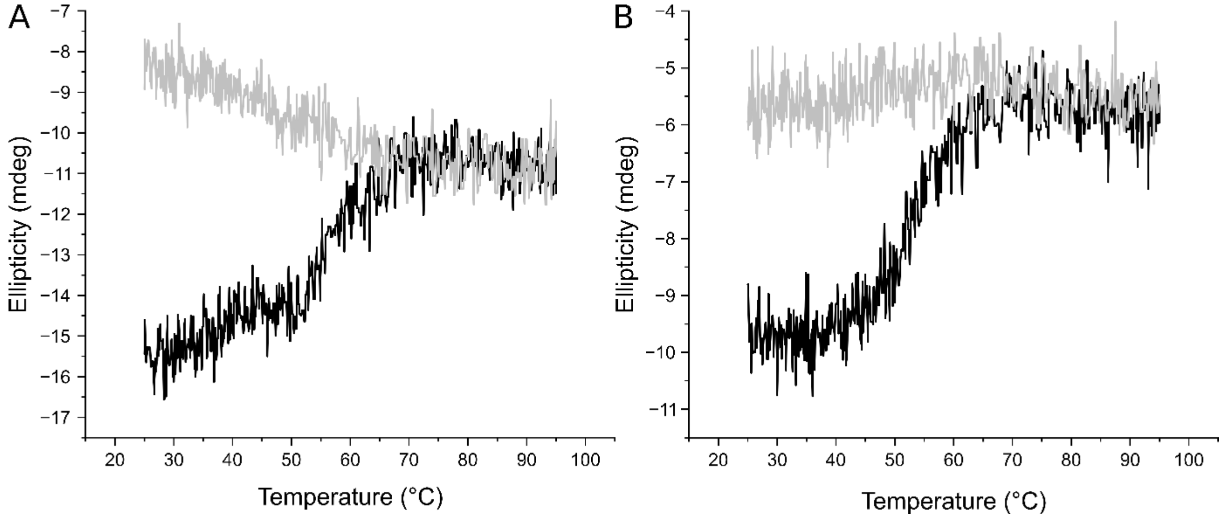

Figure S2. Thermal denaturation curves of StxA2a-His (**A**) and StcB2a-His (**B**). Signal were recorded at a Jasco J715 CD spectrometer with a PTC-348 WI Peltier unit (Jasco, Pfungstadt, Germany) at 218 nm between 25 °C and 95 °C with 1 °C/min heating rate, 0.1 °C data pitch, 1 s response time, and 1 nm band width. Black curves represent the change in signal upon heating and gray curves during the cooling process.

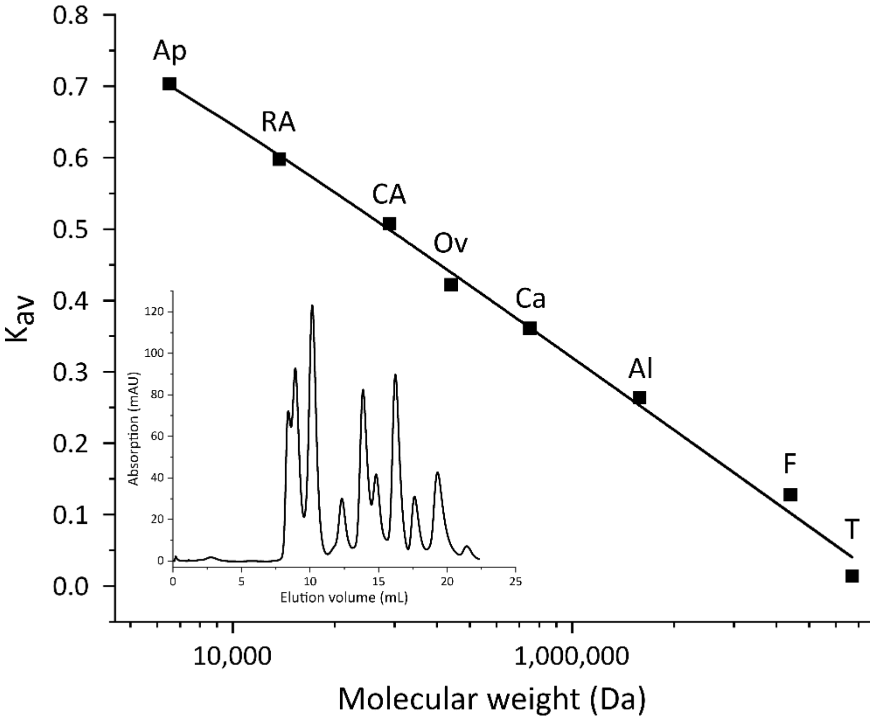

Figure S3. Logarithmic calibration curve for the Superdex® 200 increase column for the evaluation of the size exclusion experiments. Depicted are the K_{av} values against the molecular weight of the used calibration proteins. Calibration was performed using two sets of calibration runs. First run consists of the proteins thyroglobulin (T), aldolase (Al), ovalbumin (Ov), and ribonuclease A (RA) and the second run of ferritin (F), conalbumin (Co), carbonic anhydrase (CA), and aprotinin (Ap). A representative sum of the elution profiles of the calibration runs is depicted in the inlay.

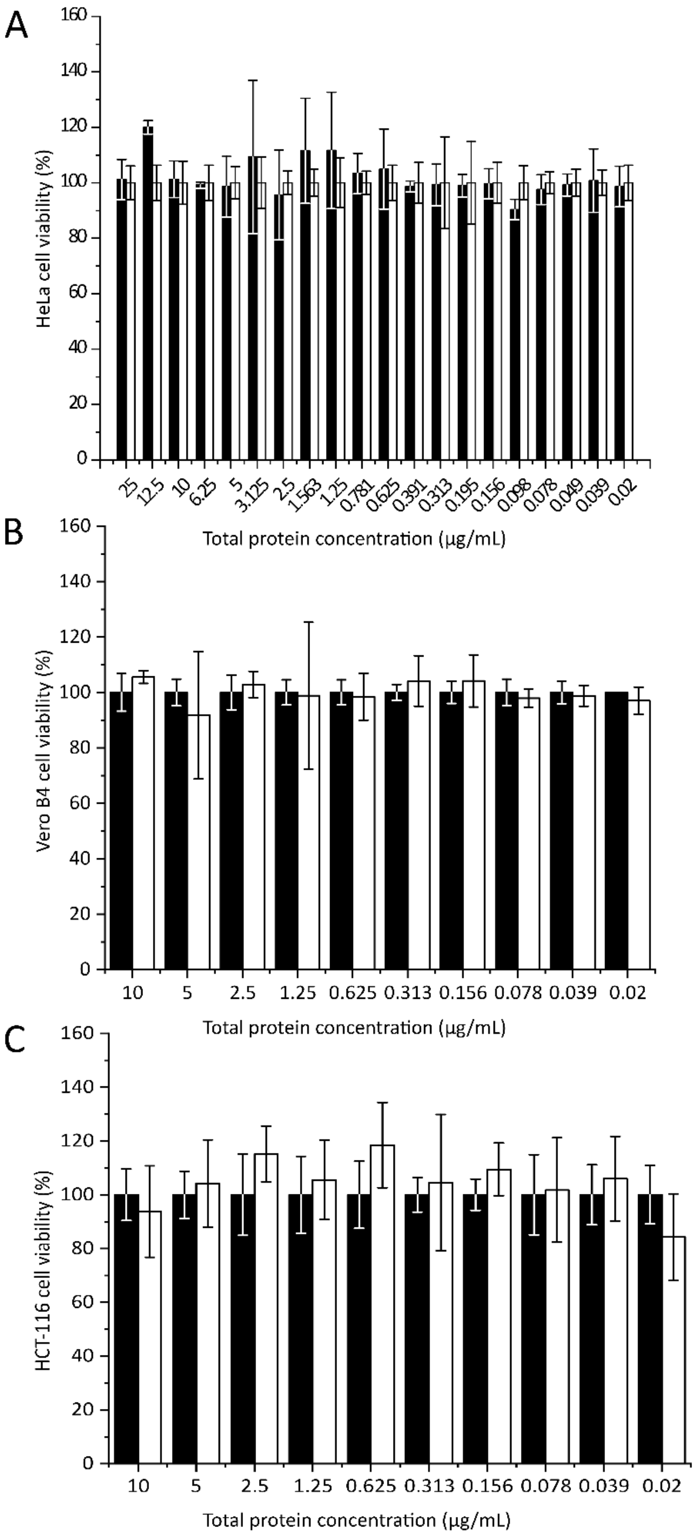

FigureS4. Cytotoxic effect of StxB2a-His on HeLa (**A**), Vero B4 (**B**), and HCT-116 (**C**) cells. Cell viabilities after intoxication with Dulbecco's phosphate buffered saline (DPBS, control, black bars), or StxB2a-His (white bars) are shown. Data of at least three biological replicates are depicted. Cell viability is shown in correlation to total protein concentration in case of StxB2a-His, values of DPBS are values dilutions series measured.

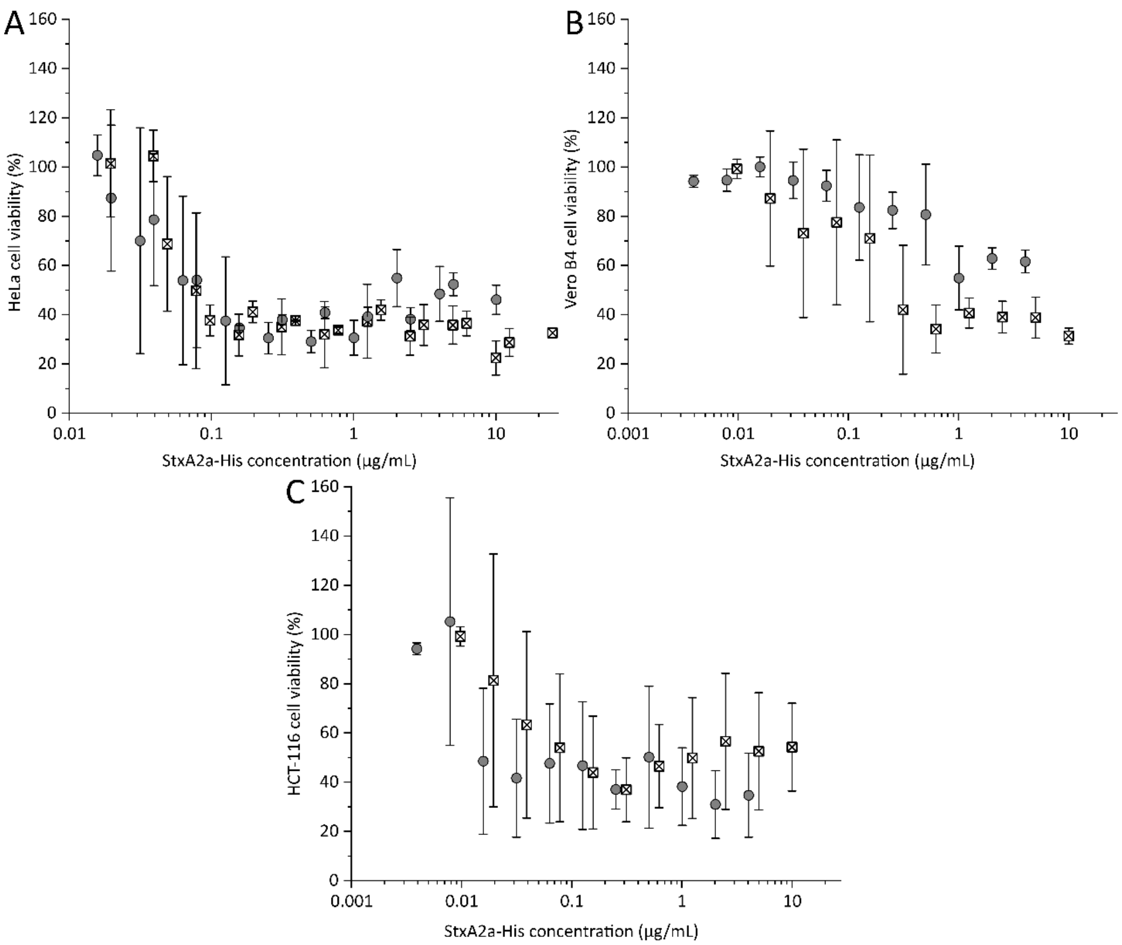

Figure S5. Cytotoxic effect of StxA2a-His on HeLa (**A**), Vero B4 (**B**), and HCT-116 (**C**) cells. Cell viabilities after intoxication with StxA2a-His in a molar ratio of 1:5 with StxB2a-His (StxAB2a-His, light grey dots), and StxA2a-His (crossed squares) are shown. Data of at least three biological replicates are depicted. Cell viability is shown in normalization to StxA2a-His concentration present in toxin solutions. Error bars represent standard deviations.

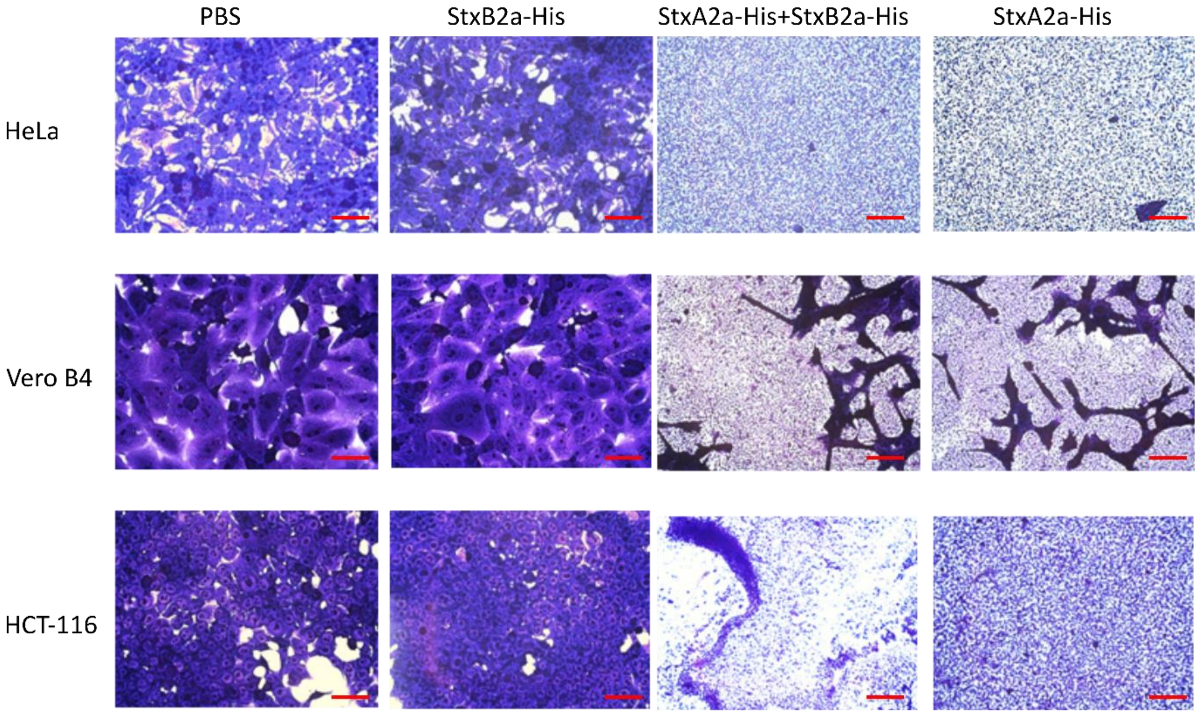

Figure S6. Microscopic analysis of cytotoxic effect of StxA2a-His on HeLa, Vero B4 and HCT-116 cells. Cells grown in 8-well Nunc permanox chambers and incubated with Dulbecco's phosphate buffered saline (PBS, control), StxB2a-His, StxA2a-His in a molar ratio of 1:5 with StxB2a-His (StxAB2a-His), and StxA2a-His are shown after 48 h incubation at 37 °C, 5.0% CO_2. Cells were stained by crystal violet staining. Data of three biological replicates is shown representatively. Scale bar: 50 μm.
